# Exceptional preservation of eye structure in arthropod visual predators from the Middle Jurassic

**DOI:** 10.1038/ncomms10320

**Published:** 2016-01-19

**Authors:** Jean Vannier, Brigitte Schoenemann, Thomas Gillot, Sylvain Charbonnier, Euan Clarkson

**Affiliations:** 1 Université Lyon 1, UMR 5276 du CNRS, Laboratoire de Géologie de Lyon: Terre, Planètes, Environnement, Bâtiment GEODE, 2, rue Raphaël Dubois, 69622 Villeurbanne, France; 2 Department of Neurobiology/Animal Physiology, Biocenter Cologne, Institute of Zoology, University of Cologne, Zülpicherstrasse 47b, D-50674 Köln, Germany; 3 Institute of Biology Education (Zoology), University of Cologne, Herbert Lewinstrasse 2, D-50931 Köln, Germany; 4 Centre de Géosciences, MINES-ParisTech, 33, rue Saint Honoré, 77300 Fontainebleau, France; 5 Muséum National d'Histoire Naturelle, Centre de Recherche sur la Paléobiodiversité et les Paléoenvironnements (CR2P, UMR 7207), Sorbonne Universités-MNHN, CNRS, UPMC-Paris6, Case postale 38, 57 rue Cuvier, F-75005 Paris, France; 6 University of Edinburgh, School of Geosciences, King's Buildings, West Mains Road, Edinburgh EH9 3JW, UK

## Abstract

Vision has revolutionized the way animals explore their environment and interact with each other and rapidly became a major driving force in animal evolution. However, direct evidence of how ancient animals could perceive their environment is extremely difficult to obtain because internal eye structures are almost never fossilized. Here, we reconstruct with unprecedented resolution the three-dimensional structure of the huge compound eye of a 160-million-year-old thylacocephalan arthropod from the La Voulte exceptional fossil biota in SE France. This arthropod had about 18,000 lenses on each eye, which is a record among extinct and extant arthropods and is surpassed only by modern dragonflies. Combined information about its eyes, internal organs and gut contents obtained by X-ray microtomography lead to the conclusion that this thylacocephalan arthropod was a visual hunter probably adapted to illuminated environments, thus contradicting the hypothesis that La Voulte was a deep-water environment.

## Abstract (web summary)

Understanding how ancient animals perceived their environment is difficult due to a lack of fossilized eye structures. Here, the authors reconstruct the compound eye of a 160-million-year old thylacocephalan arthropod, *Dollocaris*, finding evidence of hunting adaptations.

## Graphical abstract

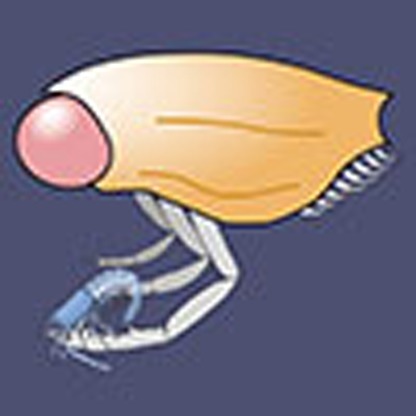

Vision is one of the key innovations that revolutionized the way animals explored their environment and interacted, with profound implications for their dynamics (for example, phototaxis), reproductive behaviour (for example, mate recognition) and feeding strategies (for example, predation^1^). The interplay between prey recognition and predator avoidance driven by visual stimuli is thought to have been a crucial factor that triggered a critical selection pressure leading to the accelerated evolution of sensory systems and defensive structures among early animal communities^2^. Fossil Lagerstätten of Cambrian age indicate that numerous early arthropods did have sophisticated compound eyes^3^,^4^,^5^,^6^,^7^ with an external faceted network often strikingly similar to that of modern crustaceans and insects. However, underlying cellular features of major importance in light reception and processing are almost never fossilized making it impossible to characterize the visual performances of these early animals and their descendants. No arthropod visual system preserved in its entirety occurs in the fossil record except that of a modern-type fly embedded in Eocene amber^8^. Receptor cells were recognized in the eyes of Devonian trilobites^6^ but more in-depth investigations are necessary to obtain a detailed reconstruction of these possible apposition-type visual structures. Here, we generate the first full, three-dimensional reconstruction of an apposition eye from external optics to internal cell receptors, in *Dollocaris*, a 160-million-year-old thylacocephalan arthropod from the Middle Jurassic La Voulte Lagerstätte (Ardèche, France^9^,^10^). The huge frontal eyes that characterize *Dollocaris* had ca 18,000 juxtaposed ommatidia each with a corneal lens, a crystalline cone and elongated receptor cells clustered around a central rhabdom. This is one of the largest number of ommatidia known in extinct and extant arthropods. These large apposition eyes have direct analogues in modern crustaceans such as amphipods^11^,^12^, and in insects such as dragonflies. Their size, panoramic field of vision, extremely high number of ommatidia and visual parameters suggest a highly resolving mosaic-like image-vision and the ability to detect and track moving objects. Additional fossil evidence such as a set of three powerful prehensile appendages and guts containing the undigested remains of mobile prey indicate that *Dollocaris* was a visual predator. Although apposition eyes most certainly evolved much earlier than the Jurassic, we present direct evidence here that the internal organization of the most common modern eye type already existed 160 million years ago.

With their huge eyes and long prehensile appendages, thylacocephalans are among the most intriguing arthropods of the Palaeozoic and Mesozoic eras (Fig. 1). Their anatomy, mode of life and phylogenetical affinities have remained largely unresolved^13^,^14^,^15^,^16^,^17^. Thylacocephalans have a remote ancestry dating to the Silurian^18^ and possibly earlier^16^ and became extinct in the late Cretaceous. They are characterized by a segmented body protected by a sclerotized ‘bivalved' carapace, a pair of large compound eyes and three pairs of powerful head appendages with chelate or spiny tips converging towards the mouth (Fig. 1d). They recall the raptorial appendages of extant mantis shrimps (stomatopod crustaceans) and suggest predatory and hunting habits. X-ray microtomography (XTM) and thin-sectioning of exceptionally well-preserved specimens from the Middle Jurassic La Voulte Lagerstätte have revealed the anatomy of Mesozoic thylacocephalans with unprecedented resolution, as exemplified here by *Dollocaris*. We also describe here, although briefly, some of the vital organs of this animal that provide precise and new information about its lifestyle (Fig. 1 and Supplementary Figs 1 and 2). *Dollocaris* had eight pairs of gills attached ventrally to the trunk segments, each bearing regularly spaced lamellae and axial afferent and efferent canals, similar to the phyllobranchiate gills^19^ of modern decapods. A tubular dorsal heart with a myocardium, a pericardial cavity and efferent arterial vessels attests to the presence of a complex haemolymph circulatory system complemented by haemolymph circulation and gaseous exchange through the integumental network of the carapace^20^,^21^. This integrated system offering enhanced exchange surfaces suggests high oxygen requirements consistent with an active lifestyle. The abdominal region bore eight pairs of very short pleopod-like appendages that protruded through the posteroventral gape of the carapace and presumably functioned in relation to swimming and ventilation. XTM also revealed the three-dimensional morphology of the digestive system. The anterior gut had a vast medial chamber (stomach) connected to the mouth via a short tubular oesophagus and flanked by two lateroventral pouches of equal size. The mouth opened ventrally between the lateral eyes (Fig. 1b,c,h). A structure with deep infoldings comparable to the pylorointestinal valve of modern decapods^22^ was present at the boundary between the stomach and the midgut. A well-developed hepatopancreas also attached in this region via symmetrical primary ducts branched off into numerous blind tubules (Supplementary Fig. 1). The intestine appears (XTM) as a long and relatively narrow tube. Resemblances to the digestive system of modern crustaceans suggest a relatively complex food processing chain that consisted of: (i) food storage in the stomach evidenced by preserved food contents (for example, exoskeletal elements of small crustaceans; Fig. 1k,l), (ii) transfer of finer and fluidized food into the hepatopancreas where assimilation also took place and (iii) excretion of waste products via the intestine. The relatively large undigested exoskeletal fragments that were found in the medial chamber (stomach) are unlikely to have transited throughout the narrow intestine and were most probably regurgitated by the animal. The huge eyes of thylacocephalans have long been a subject of debate^13^,^23^ but have neither been described in detail nor interpreted via comparisons with recent visual models.

Thylacocephalans have been tentatively assigned to a great variety of crustacean groups (for example, stomatopods, decapods, cirripeds) as they were first described. More recently, Haug *et al.*^18^ have proposed a sister-group relationship with Remipedia based on morphological similarities between *Thylacares* from the Silurian Waukesha fauna from Wisconsin and extant remipeds. These resemblances concern the multisegmented and undifferentiated nature of the trunk *sensu lato* and the number (3) and detailed morphology of the sub-chelate raptorial appendages. This tentative placement of Thylacocephala close to remipeds is questionable given major differences between the two groups in terms of body plan and exoskeletal design. For example, extant remipeds such as *Speleonectes*^24^ have an elongated, flexible body with up to 36 trunk segments, a uniform series of laterally inserted trunk appendages and a very short head shield, a set of characters that are found in no Palaeozoic (for example, *Thylacares*^18^) or Mesozoic (for example, *Dollocaris*, this paper) thylacocephans. The affinities of thylacocephalans will not be resolved until we obtain precise information on their anterior appendages (for example, antennae, mandibles) and thereby establish homologies with the appendages of extant arthropods/crustaceans. Although phylogenetic analysis lies out of the scope of the present paper, our detailed study of *Dollocaris* highlights important features of great interest to the phylogeny of Thylacocephala. *Dollocaris* has three well-defined tagmata: a head bearing apposition eyes and at least three pairs of raptorial appendages, a trunk with eight pairs of gills and an abdomen with eight segments bearing short pleopods. Although much uncertainty remains concerning possible additional head appendages^18^, this body plan recalls that of eumalacostracans (5+8+6) and Phyllocarida (5+8+7), which would support the hypothesis of malacostracan affinities for Thylacocephala. Thylacocephala as a possible malacostracan stem-group is a relevant hypothesis to explore through future cladistic analyses.

In summary, X-ray tomography combined with electron microscopy reveals unknown aspects of the biology of Mesozoic thylacocephalan arthropods such as their digestive system, their gut contents and their visual organs, with unprecedented accuracy. The internal structure of the large apposition eyes of *Dollocaris* is reconstructed and indicates that this arthropod had an acute vision adapted to illuminated environments. Its habitat and behaviour are interpreted in the light of its assumed visual performances and palaeoenvironmental evidence. *Dollocaris* was most probably an ambush visual predator.

## Results

### Reconstructing the eye structure of *Dollocaris*

**Figure 1:**
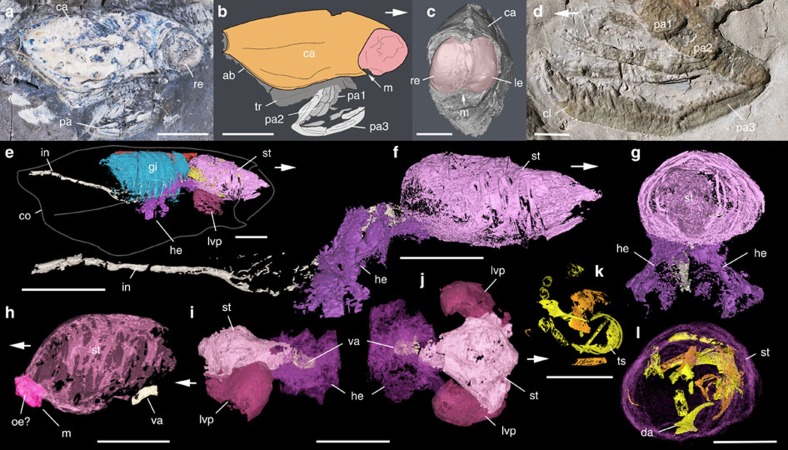
External and internal morphology of *Dollocaris ingens*. Thylacocephala; Middle Jurassic La Voulte Lagerstätte. (**a**,**b**) MNHN.F.R50939, right lateral view. (**c**,**h**) MHNL-20293244, frontal view showing the pair of bulbous eyes and details of anterior part of digestive system in lateral view. (**d**) MNHN.F.R06202, details of prehensile appendages. (**e**–**g**,**k**,**l**) UJF-ID-1799, XTM reconstructions of internal anatomy (respiratory and digestive system), general view and details of digestive system in lateral and anterior views, and crustacean exoskletal fragments inside the stomach. (**i**,**j**) FSL 710067, XTM reconstructions of the anterior part of digestive system in lateral and dorsal views. White arrows indicate front part. ab, abdomen; ca, carapace; cl, claw; co, carapace outline; da, dactylus; gi, gills; he, hepatopancreas; in, intestine; le, left eye; lvp, latero-ventral pouch; m, mouth; oe?, possible oesophagus; pa1-3, 1st to 3rd pair of prehensile appendages; re, right eye; st, stomach; tr, trunk; ts, trunk sclerite (fragment); va, valve. Scale bars, 10 mm.

**Figure 2:**
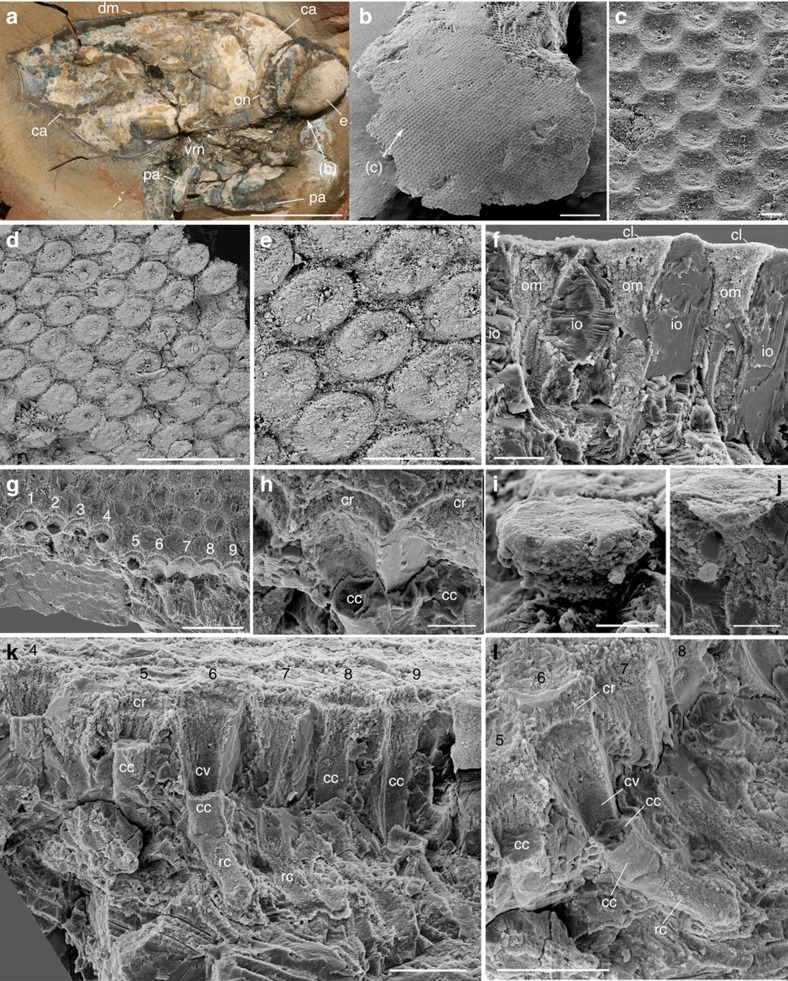
Eye structure of *Dollocaris ingens*. Thylacocephala; Middle Jurassic La Voulte Lagerstätte. (**a**) FSL 710064, lateral view of a complete specimen preserved in a nodule showing large frontal eye. (**b**,**c**) Fragment of eye with faceted hexagonal pattern, general view and details. (**d**,**e**) Eye surface with corneal lenses, general view and details. (**f**) Section through eye showing three ommatidia separated by mineralized inter-ommatidial material. (**g**,**h**) Nine ommatidial cavities, general view and details of ommatia 5–7. (**i**,**j**) Disk-like corneal lens. (**k**,**l**) Section through eye showing ommatidial structure (ommatidia 4–9 as in **g**). FSL 710064 in **a**–**e**,**g**–**l**; MNHN.F.A29278 in **f**. All SEM images except **a**. **d** and **e** are back-scattered images. ca, carapace; cc, crystalline cone; cl, corneal lens; cr, corneal rim; cv, cavity of crystalline cone; dm, dorsal margin; e, eye; io, inter-ommatidial material; om, ommatidium; on, carapace optic notch; pa, prehensile appendage rc, retinula cells (sensory cells); vm, ventral margin; 1–9, ommatidia 1–9. Scale bars, 10 mm in **a**, 500 μm in **b**, 100 μm in **d**,**g**, 50 μm in **e**,**k**,**l**, 20 μm in **c**,**f**,**h**–**j**.

**Figure 3:**
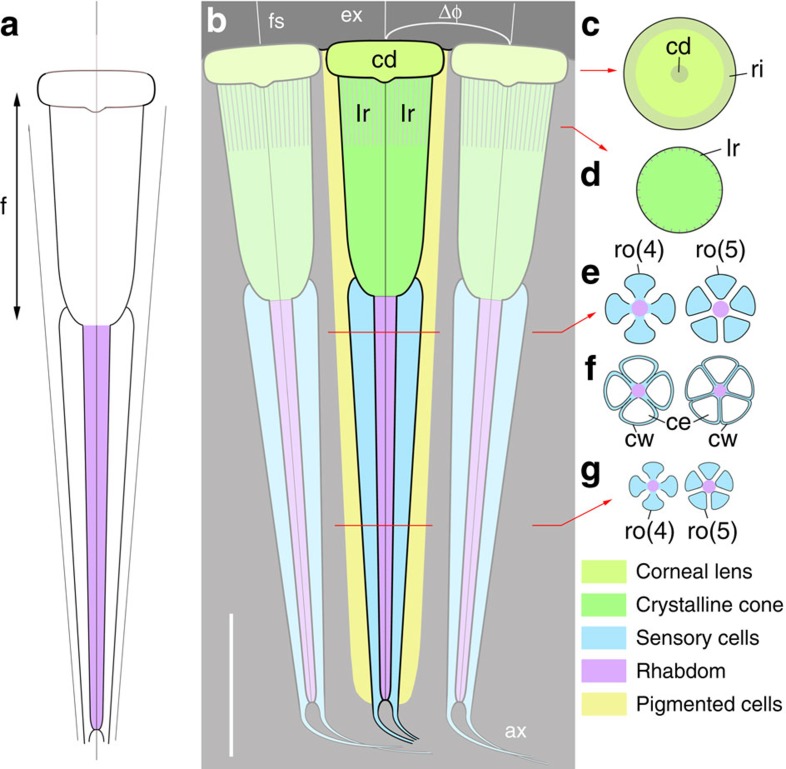
Reconstruction of the eye structure of *Dollocaris.* Thylacocephala; Middle Jurassic, showing three adjacent ommatidia. The most distal disk-like feature is interpreted as the corneal lens. The underlying crystalline cone is assumed to form an image at its proximal tip in direct contact with the rhabdom. The 4 or 5 retinula cells form a long tapering cylindrical feature that extends into nerve fibres. In transverse sections, the retinula cell clusters appear as a rosette-like structure with the central axis probably representing the rhabdom. Cellular details of the inter-ommatidial region are not preserved except possible pigmented areas along the crystalline cone (Supplementary Fig. 3). (**a**) Simplified ommatidium. (**b**–**g**) Simplified longitudinal (**b**) and transverse sections through ommatidia with 4 and 5 retinula cells and two types of cellular preservation (cells empty with mineralized cellular walls or cells mineralized as a whole). ax, axonal structure; cd, central depression; ce, cell; cw, cell wall; d, rhabdom diameter; Δ*φ*, inter-ommatidial angle; ex, external medium; *f*, focal length; fs, faceted surface; lr, longitudinal ridge; ri, rim; ro(4), rosette-like structure with 4 cells; ro(5), rosette-like structure with 5 cells. Scale bar, 50 μm.

**Figure 4:**
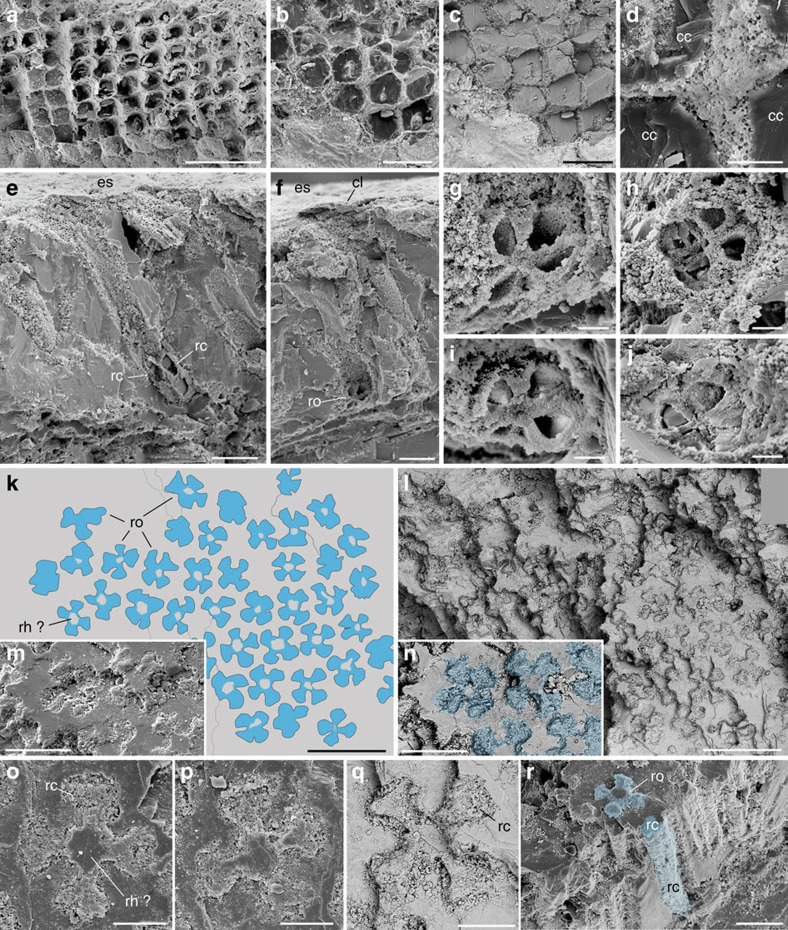
Internal structure of the eye of *Dollocaris ingens.* Thylacocephala; Middle Jurassic La Voulte Lagerstätte. (**a**–**d**) Transverse sections through adjacent ommatidia showing juxtaposed crystalline cones, general view, details and interspace between crystalline cones. (**e**) Longitudinal section through ommatidia showing two retinula cells. (**f**,**g**) Transverse section through a rosette-like cluster of 4, possibly 5 retinula cells, general view and details. (**h**–**j**) Details of rosette-like clusters with 4 retinula cells. (**k**–**n**) Transverse section through the retinula cells of numerous ommatidia, general view, simplified drawing (cells in blue) and details. (**o**–**q**) Details of rosette-like structures. (**r**) Rosette-like structure and elongated three-dimensional structure of a retinula cell. FSL 710064 in **a**–**j**, MNHN.F.R06206 in **k**–**r**. All SEM images. **c**,**l**,**n**,**q** are back-scattered images. cc, crystalline cone; cl, corneal lens; es, eye external surface; rc, retinula cell; rh?, possible rhabdom; ro, rosette-like structure (section through cluster of retinula cells). Scale bars, 100 μm in **a**,**k**,**l**; 50 μm in **b**–**d**,**m**,**n**,**r**, 20 μm in **e**,**f**,**o**–**q**, 5 μm in **g**–**j**.

Most Mesozoic thylacocephalans had a frontal pair of bulbous compound eyes with an extremely large visual surface (Fig. 1a–c). The eye of *Dollocaris ingens* reaches up to almost one fourth of its body length. Comparable visual hypertrophy is rare in modern arthropods with the exception of some deep-sea hyperiid crustaceans^12^ and to a lesser extent dragonflies. The exceptionally fine calcitic and phosphatic preservation of *Dollocaris* eyes from La Voulte allows comparisons at the cellular level with the eyes of modern arthropods^25^,^26^. The *Dollocaris* eye surface displays a dense, regular, hexagonal pattern of facets (Fig. 2b–e) as in numerous modern insects and crustaceans, with an estimated density of more than 500 ommatidia per mm^2^ (Supplementary Note 1). This means that each eye (for example, see Fig. 2a) would have borne more than 18,000 ommatidia. Various longitudinal and transverse sections through the eye allowed detailed three-dimensional observations of the whole ommatidium structure. Each facet accomodates a flattened, lens-like structure of ∼40 μm in diameter and 15 μm in thickness that is nested in a slightly larger circular rim (Figs 2f–j and 3). This is the most external feature of the ommatidium, interpreted as the possible corneal lens. Its external surface is often slightly concave because of the possible post-mortem collapse and/or shrinkage of the original structure. A small depressed area or pit is frequently observed in the centre of the lens (Fig. 3d,e). Below it is a columnar tapering feature with sharp boundaries. Its diameter varies from ∼20 μm to nearly 40 μm at the contact with the corneal lens, and its total length approaches 80 μm (Figs 2k,l and 3 and Supplementary Fig. 3a–g). This bullet-shaped feature most likely represents the crystalline cone, an important component of modern aquatic arthropod eyes that forms part of the dioptric apparatus and refracts incoming light into the receptor region. The crystalline cone of *Dollocaris* is preserved in the form of either large (possibly single) crystals of apatite with a glassy appearance (Fig. 4b–d) or microcrystalline phosphatic material (Fig. 2f; energy dispersive X-ray spectroscopy (EDX) analysis, Supplementary Fig. 4). The upper third of the outer wall of the cone appears to be striated with tiny longitudinal ridges equidistant and ∼3 μm apart. The crystalline cone is followed by an elongated, tapering and often curved feature of at least 130–150 μm in length (Figs 2k–l and 3 and Supplementary Fig. 3a,g). Transverse sections through it reveal four or five triangular structures arranged in a rosette-like pattern around the longitudinal axis of the ommatidium (Fig. 4g–r). These rosettes have the same basic arrangement as the retinula cells of numerous modern arthropods (Supplementary Figs 5 and 6) and are interpreted as such, thus confirming previous hypotheses^23^. In extant arthropods, retinula cells are specialized neurons that form a rod-like structure centred on the optical axis just below the crystalline cone^25^. This is the receptor region of the arthropod eye. The assumed retinula cells of *Dollocaris* show two types of mineralization: (ii) the microcrystalline coating of the cellular membranes that left cytoplasmic cavities empty (triangular hollows; Fig. 4g–j) or (ii) the complete infilling of the cells by microcrystalline apatite (filled rosette-like structures; Fig. 4o–q). A cylindrical axis (diameter between 5 and 15 μm) occurs in numerous rosettes (Fig. 4o) and may correspond to the rhabdom of modern arthropod eyes where light-sensitive pigments are stored. The overall size of the rosettes and the diameter of their axis depend on their location along the receptor cells unit (Fig. 3). The exact manner in which the retinula cells cluster terminates is unclear. However, these cells seem to taper into thinner closely packed oblique structures that underlay the receptor region (Supplementary Fig. 3d,g). These structures might constitute the axonal network. Each visual unit is separated by a relatively thick inter-ommatidial material typically composed of calcite (EDX analysis, Supplementary Fig. 4) in which cellular details are not discernable. This space is likely to have been occupied by various pigmented cells that normally surround the whole ommatidium of modern arthropods.

## Discussion

The eyes of *Dollocaris* show no interval separating the crystalline cone from the underlying sensory features as is the case in superposition eyes^26^,^27^. On the contrary, each ommatidium has a crystalline cone that seems to be in contact with the receptor cells. This is a diagnostic feature of compound apposition eyes. Along with an Eocene dolichopodid fly^8^, *Dollocaris* has the best preserved fossil eyes ever described for an arthropod, exhibiting exquisite details from the corneal surface to axonal features that enable detailed comparisons with their modern counterparts. The eyes of this Eocene fly belong to the superposition type thus differing from those of *Dollocaris*. The number of receptor cells in modern insects and crustaceans varies between groups, from 4 to 6 in non-malacostracan groups to typically 8 in insects and malacostracans, to a maximum of 11 in some amphipods^26^. These cells always form a rosette-like structure. Hyperiid crustaceans such as *Phronima* have five retinula cells^28^ (Supplementary Fig. 6a–c) that, when seen in transverse sections, display a regular rosette-like pattern strikingly similar in size (ca 25 μm) and shape with that observed in *Dollocaris* (Fig. 3). In most modern crustaceans and insects, the inner part of the retinula cells fuse into the rhabdom, a slender, rod-like receptive structure that consists of interdigitating microvilli where light-sensitive pigments are stored. The *Phronima* rhabdom is circular or pentagonal in transverse section^28^ (Supplementary Fig. 6b,c). The rosette-like features of *Dollocaris* have a central area of about the same diameter (5–10 μm) as the rhabdom of *Phronima* and other crustaceans (Fig. 4o–q and Supplementary Fig. 6). However, it is unclear whether this mineralized central axis represents the rhadom of *Dollocaris* or is an artefact. Groups of five retinula cells are known in other hyperiids such as *Streetsia*^29^ and more generally in other amphipods^30^. The *Dollocaris* ommatidium has a well-developed crystalline cone capped with a corneal lens as in numerous malacostracan crustaceans and insects. Non-malacostracan crustaceans lack a corneal lens^26^. Other important characteristics of modern apposition eyes are unfortunately not discernible in *Dollocaris* such as the number of cone cells (four in insects and malacostracans) and the cellular structure of the inter-ommatidial space. Different types of pigment cells, including a pair of primary pigment cells occur in the apposition eyes of extant insects and crustaceans, their main function being to prevent the spread of light to neighbouring ommatidia. Our fossil specimens reveal no such cellular details that might have been obliterated by early diagenetic mineralization (Fig. 2f and Supplementary Fig. 4). It appears that the rosette-like structures formed by the retinula cells are shielded by imbricated elongated units (possibly 6; Supplementary Fig. 5i,j) that may represent such pigmented cells. Aligned and clustered microspherical hollows (diameter ca 0.5 μm; Supplementary Fig. 3h–j) might represent the remnants of possible pigment granules (melanosomes).

Measuring optical features and calculating visual parameters can be easily achieved with biological specimens (for example, extant insects and crustaceans^31^,^32^,^33^,^34^,^35^,^36^,^37^) but becomes much more challenging with fossils such as *Dollocaris*. Although the eyes of *Dollocaris* show extremely fine details of their internal structure (for example, receptor cells), these are often incomplete, displaced or deformed with their ommatidial layer tilted, because of the post-mortem collapse of tissues and taphonomic factors. These unfavourable conditions do not allow direct accurate measurements of key parameters such as the bending radius, the rhabdom diameter, the inter-ommatidial angle and the rhabdom acceptance angle. We are nevertheless able to make, in some cases, approximations with acceptable accuracy, our final objective being to propose a sound interpretation of the function of the huge eyes of *Dollocaris* in relation to its lifestyle and assumed habitat.

Each compound eye of *Dollocaris* has an exceptionally high number of ommatidia that can be estimated to ∼18,000 on the basis of facet countings in well-preserved specimens and assuming that each eye is approximately an hemisphere with evenly distributed ommatidia (Supplementary Note 1 and Supplementary Fig. 7). This remains a rough estimate that does not take into account possible local variations of the bending radius and the ommatidial size (for example, acute zone of some modern insects and crustaceans)^32^,^38^,^39^,^40^,^41^,^42^,^43^,^44^. Each hemispherical eye had a wide field of view providing the animal with a panoramic vision (Fig. 1 and Supplementary Fig. 7a–f). Such remarkable attributes are known in some modern insects and crustaceans that evolved large faceted eyes, high ommatidial densities and a panoramic field of vision mainly in relation to their hunting behaviours^31^,^38^,^45^,^46^. Typically, dragonflies such as *Anax* have ca 30,000 facets per eye^47^, praying mantis 9,000 (ref. 48) and some mantis shrimps up to 10,000 (for example, *Odontodactylus*^49^). Hyperiid amphipods also belong to the category of visual predators with hypertrophied, panoramic eyes resembling those of Mesozoic thylacocephalans (Supplementary Fig. 6a) but they have fewer and larger ommatidia (for example, 750 in *Phronima*^50^). Moreover, numerous hyperiids show a dorso-ventral eye differentiation (for example, with larger dorsal facets^12^) and other specialized internal features that are not observed in *Dollocaris* whose facet diameter (*D*) seems to be virtually constant (ca 40 μm) at least laterally. Compound eyes with small lens diameter require high light intensities in order to capture enough signal from photons and are typically found in arthropods living in shallow waters and with a diurnal habits. Typical examples are bees (*D*=25 μm (ref. 40)), mosquitoes (20<*D*<29 μm (ref. 51)), the mantid *Tenodera* (*D*=35 μm (ref. 52)) the crab *Leptograpsus* (22<*D*<50 μm (ref. 53)) and the shallow water shrimp *Palaemonetes* (*D*=50 μm (ref. 27)). Similarly, the facets of *Dollocaris* (*D*=40 μm) would point to a comparable adaptation to high light intensities.

The ability of an eye to resolve details depends on the angular separation between its receptors^36^,^50^. In an apposition eye, it is the inter-ommatidial angle (Δ*φ*) that determines how the overall image is sampled. Its acuity is inversely proportional to Δ*φ* but the overall performance of the eye also largely depends on the optical quality of the ommatidia, the rhabdom dimensions and the amount of light available^54^. Measuring Δ*φ* involves techniques (for example, histology, pseudopupils) that cannot be used in our fossil specimens. We are using here an estimation method proposed by Land^54^ that when applied to the hemipherical eyes of modern insects gave estimates close to measured values. We obtain for *Dollocaris* a predicted value of Δ*φ* close to 1° (see details in Supplementary Note 1). In most common flying insects Δ*φ* ranges between 1 and 3° (ref. 54).The smallest inter-ommatidial angle in extant arthropods occurs in dragonflies such as *Anax junius* (Δ*φ=*0.24° in the acute zone^54^). Their remarkable acuity makes them able to detect objects from a greater distance and to track prey with great efficiency. Comparable acute apposition eyes are known in some modern midwater/deep-sea amphipod crustaceans such as *Phronima* (Δ*φ=*0.4° in the dorsal acute zone^54^). With Δ*φ* of ∼1°, the eye of *Dollocaris* has the characteristics of an acute eye.

The field of view of each ommatidium is another important visual parameter that can be defined by the angle (Δ*ρ*) subtended by the rhabdom tip at the nodal point of the corneal lens^25^. The rhadom is a rod-like structure in the centre of each ommatidium, composed of microvilli extending from the surrounding retinular cells. Calculating Δ*ρ* requires accurate measurements of the rhabdom diameter (*d*). Whereas the outlines of the receptor cells of *Dollocaris* are well-defined in transerve sections (rosettes; Fig. 4), those of the rhabdom are indistinguishable. Transverse sections through the ommatidia often display a well-defined central area that may correspond to the rhabdom (see Fig. 4o–r and Supplementary Fig. 5h–j for details) but major uncertainty remains as to the exact nature of this axial feature (taphonomic artefact?). The light sensitivity of an apposition eye (*S*) expresses its ability to absorb photons from a standardized light source. It is controlled by the lens diameter (*D*) and Δ*ρ*. The impossibility of estimating Δ*ρ* precludes any prospect of evaluating the light sensitivity of the eye of *Dollocaris*. However, the relative dimensions of its ommatidial components (relatively small facet diameter, long focal length and small diameter of rhabdomere unit; Fig. 3) seem to be poorly consistent with a high light sensitivity. Typically, the deep-sea isopod *Cirolana*^55^ owes its high sensitivity to its huge corneal lens and very large rhabdom diameter. We assume that the eye of *Dollocaris*, in order to function in optimal conditions, must have required a large to moderate amount of light (high number of photons).

Palaeogeographic reconstructions for the Jurassic place the La Voulte Lagerstätte along the western margin of the Tethys Ocean^56^ characterized by a complex submarine palaeotopography of tilted blocks. It was situated near the slope−basin transition^57^,^58^ in the context of the passive margin of a sedimentary basin subjected to thermal subsidence^57^. Combined geological and palaeontological evidence have been used to infer the depositional environment of the Jurassic La Voulte Lagerstätte^9^,^10^,^58^,^59^,^60^. The ca 10-m-thick unit which contains exceptionally preserved fossils is dominated by dark clayey marls containing calcareous nodules of early diagenetic origin. These fine terrigeneous clastics and the absence of storm layers and shell beds indicate that the deposition took place below storm wave base. By definition, the storm wave base is the water depth to which average storm waves can affect the sea floor and is typically 30–100 m in open seas. Although water depths exceeding 200 m have been proposed^58^, precise bathymetry cannot easily be determined. Numerous animal groups found at La Voulte such as vampyromorphid and teuthoid cephalopods^9^,^61^,^62^, cyrtocrinid crinoids^9^,^59^, pycnogonids^9^,^63^, polychelidan crustaceans^9^,^64^ have exact counterparts in the present-day deep-sea. Moreover, siliceous sponges lack the characteristics of their fossil equivalents from shallow-water bioherms. This distinctive faunal association has been used as a supporting evidence for a relatively deep-water environmental setting at La Voulte, which would imply a low illumination level. However, this hypothesis should be treated with caution considering that the bathymetrical range occupied by the modern deep-sea fauna might have resulted from downslope shifts through time.

Although the eyes of *Dollocaris* were undoubtedly acute, their ability to capture light and low sensitivity make them *a priori* better adapted to high or moderate light conditions than to the poorly illuminated environments predicted at La Voulte. Resolving this apparent paradox requires more detailed palaeoenvironmental studies and new data on the visual organs of animals (for example, other crustaceans) associated with thylacocephalans in the La Voulte exceptional biota.

**Figure 5:**
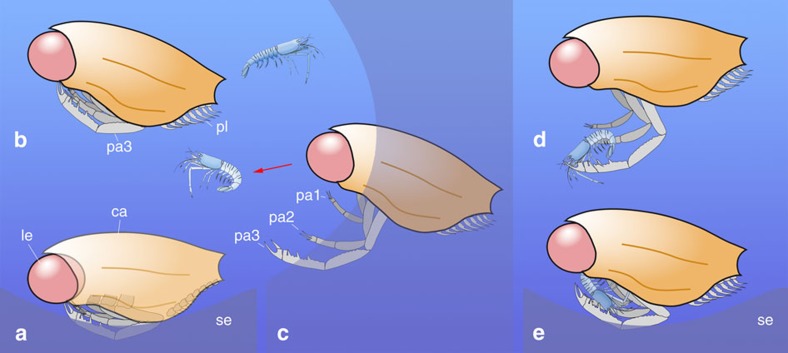
*Dollocaris* (Middle Jurassic La Voulte Lagerstätte) as a nektobenthic visual arthropod. (**a**) Resting at the water-sediment interface. (**b**) Swimming near the bottom by using its pleopods. (**c**–**e**) Detecting (red arrow) and capturing prey from a concealed position (for example, submarine relief, crevice) through a surprise attack. (**c**) Prehensile appendages (pa1-3) unfolded. (**d**) Grasping prey by means of its clawed/spiny pa1-3. (**e**) Bringing prey to the mouth via the combined action of pa1-3 and, maceration/ingestion of food at the water-sediment interface. Idealized reconstructions of the animal in left lateral view (eye in red, carapace in orange, left prehensile appendages in grey). Juvenile solenocerid shrimp in light blue (for example, *Archeosolenocera*^12^ from the La Voulte Lagerstätte) as a potential prey. ca, carapace; le, left eye; pa1-3, 1st to 3rd pair of prehensile appendages; pl, pleopods; se, sediment.

If we suppose that *Dollocaris* was visually adapted to relatively well-illuminated marine environments, then it must have inhabited the euphotic zone. Where within the water column is an important point to be discussed. *Dollocaris* was able to swim through the rhythmic beating of its short pleopod-like posterior appendages (Fig. 5). Its streamlined carapace that encapsulated most of its body features is likely to have reduced drag and minimized resistance when moving through water. However, *Dollocaris* clearly lacks the characteristics of a fast swimmer such as long swimming appendages and a flexible abdomen protruding outside the carapace. It is unlikely to have been a pelagic arthropod that lived permanently within the water column and actively migrated through it. Undigested exoskeletal elements of small crustaceans (for example, isolated dactylus and cylindrical abdominal segments of possible juvenile solenocerid shrimps^10^) found in the stomacal pocket of *Dollocaris* (XTM; Fig. 1k,l) indicate that *Dollocaris* was a visual predator that was able to spot and catch its prey within the water column. Although its hunting behaviour remains hypothetical, it is clear that its huge, panoramic, multi-faceted acute eyes were crucial to scanning its environment and to detecting potential moving prey. Directional motion computed by each eye's lobes and integrated binocularly would have been enough to trigger the rapid extension of its raptorial appendages towards the prey (Fig. 5). It seems more plausible to consider *Dollocaris* as a nektobenthic ambush predator (Fig. 5c–e) capturing prey from a concealed position (submarine relief, crevices?) through a surprise attack. Another option would be to consider *Dollocaris* as a possible migrant through the water column living alternately in relatively well lit surface waters and darker environments with visual and neural structures adapted to the respective light conditions. However, such migrations would require sufficient swimming power and buoyancy control.

Numerous arthropod eyes have evolved various strategies in order to increase light capture and obtain brighter images than is possible with conventional apposition eyes. For example, superposition eyes^65^,^66^ gain in brightness but loses in sharpness. They are found in nocturnal insects (refracting type), crabs (parabolic type) and decapod crustaceans such as shrimps, crayfish and squat lobsters (reflecting type). These eyes have a clear zone between the optical structures and the receptor cells that does not occur in *Dollocaris*. In neural superposition eyes, there is no such clear zone. Each point in space is seen by receptive elements under seven facets and their axons superimpose to a neural cartridge under the central ommatidium^67^. This type of eye is known only in dipteran insects.

Another widespread strategy to enhance sensitivity is neural pooling that can be applied to apposition eyes. For example, the apposition eyes of some nocturnal bees and wasps have an optical sensitivity 30 times greater than that of their closest diurnal relatives^68^. This visual adaptation to dim-light conditions is obtained via a sophisticated neural processing of the slow and noisy visual signals generated by the photoreceptors, which are summed by second-order monopolar cells in the lamina. The optimum visual performance of these insects can be achieved by a receptor integration time of ca 30 ms and a summation of about 12 ommatidia bundled to a cartridge^67^,^69^. In theory, optimal neural summation has the potential to make apposition eyes operational even at light intensities 100,000 dimmer than those in which they would normally perceive no light^70^. That the eyes of *Dollocaris* were equipped with such sophisticated neural networks remains purely hypothetical in the absence of fossil evidence from neural structures. However, it appears to be one of the most plausible solutions to enhance its capacity to discrimate shapes (for example, potential prey) against a poorly illuminated and noisy background, if we suppose that *Dollocaris* inhabited the lower part of the euphotic zone where light intensity is lower than in surface waters.

## Methods

### Material

The fossil material studied here comes from the early Callovian La Voulte-sur-Rhône Lagerstätte (Ardèche, SE France) and is deposited in the collections of the following institutions: Université Claude Bernard Lyon 1, Villeurbanne, France (FSL collection numbers); Musée des Confluences, Lyon, France (MHNL); Muséum National d'Histoire Naturelle, Paris, France (MNHN); Université Joseph Fourier, Institut Dolomieu, Grenoble, France (UJF-ID).

### Analyses of fossils

Specimens were observed under a Leica MZ125 stereomicroscope microscope equipped with Plan × 1.0 and Planapo × 1.6 lenses, digital camera and Leica LAS 3.7.0 imaging system with multifocus option) and photographed using a D3X-Nikon camera with Nikon Micro-Nikkor 60 lens. Scanning electron microscopy (FEI Quanta FEG 250) and EDX analysis were used (CTμ facilities at the University Lyon 1) to study the detailed morphology of the visual structures and their chemical composition. Images were acquired with Secondary Electron and Back-Scattered Electron detector*s* at 15 kV and under high vacuum.

Three-dimentionally preserved specimens of *Dollocaris* were scanned on a Metris X-Tek HMX-ST scanner (Natural History Museum, London) with a tungsten reflection target at 200 mA and 225 kV, 0.17–1 s exposure times for 3,142 projections and a 1-mm copper filter. The 4 MP (2,000 × 2,000) Perkin Elmer detector panel (Perkin Elmer) provided a voxel size (resolution) of 15–25 mm. Three-dimensional models were created from the tomographic data sets using the Materialise Mimics Innovation Suite, version 14, at the Natural History Museum, Paris.

## Additional information

**How to cite this article:** Vannier, J. *et al.* Exceptional preservation of eye structure in arthropod visual predators from the Middle Jurassic. *Nat. Commun.* 7:10320 doi: 10.1038/ncomms10320 (2016).

## Supplementary Material

Supplementary Information Supplementary Figures 1-7, Supplementary Table 1, Supplementary Note 1 and Supplementary References
