# Supplementary material for: Exceptional preservation of eye structure in arthropod visual predators from the Middle Jurassic
Source: Nat Commun. 2016 Jan 19;7:10320. doi: 10.1038/ncomms10320 (PMC4735654; doi:10.1038/ncomms10320)
Supplement: Supplementary Information — Supplementary Figures 1-7, Supplementary Table 1, Supplementary Note 1 and Supplementary References [file ncomms10320-s1.pdf]

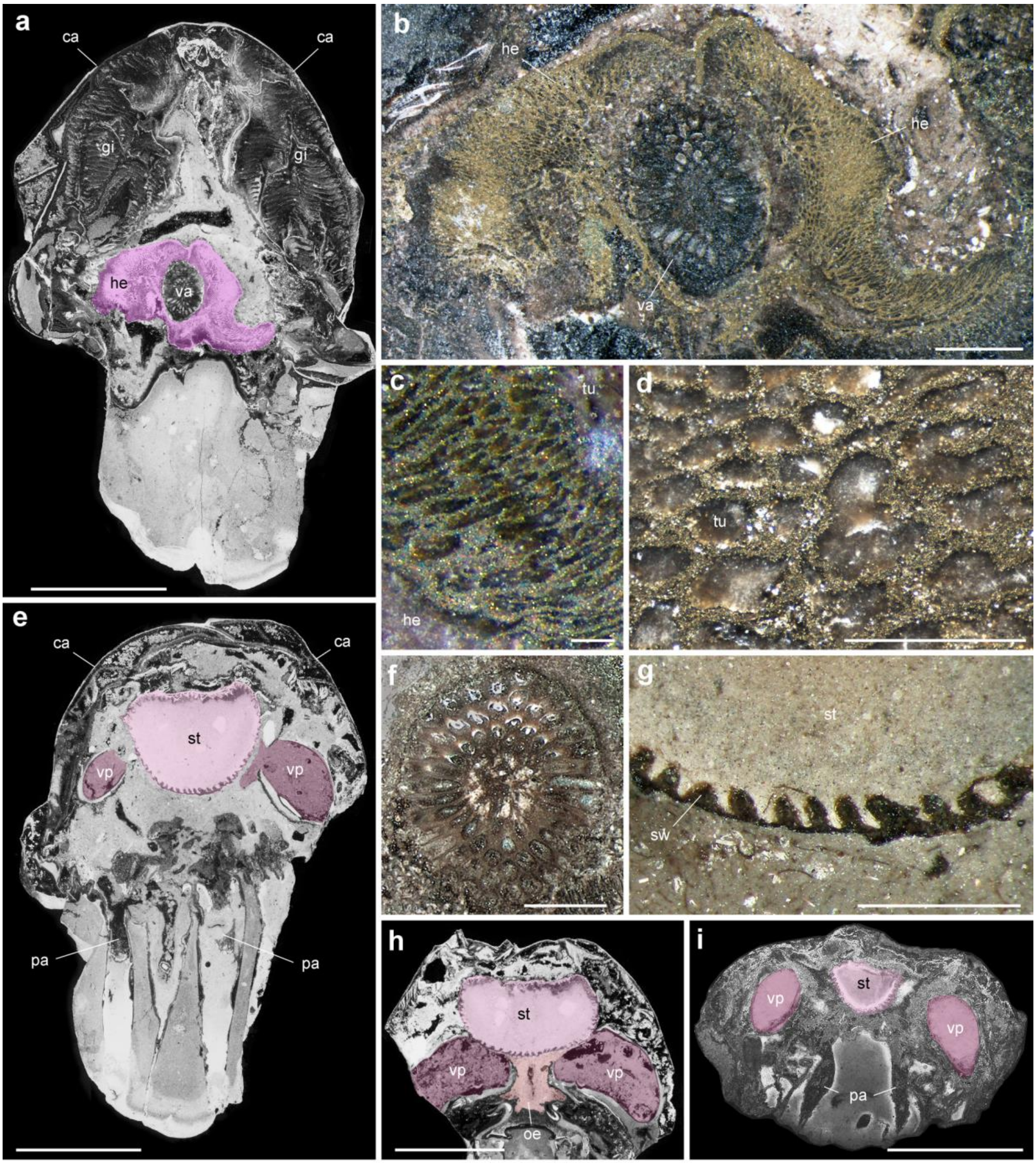

Supplementary Figure 1 | Digestive system of *Dollocaris ingens* (Middle Jurassic La Voulte Lagerstätte). **a-h**, MNHN.F.R06054, polished transverse sections through the body. **a-d**, section through the hepatopancreas (bright pink) and the valve, general view, details of the hepatopancreatic region and details of hepatopancreatic tubules (walls preserved in pyrite). **e**, section through the mid part of the stomach. **f**, section through valve at the boundary between the stomach and the midgut. **g**, details of stomach wall with deep longitudinal infoldings. **h**, section through the ventral pouches (dark pink) and the oesophagus (light orange). **i,** FSL 710104, transverse section through the anterior part of the body. All light photographs. Scale bars: 1cm in a, e, h, i; 2 mm in b, f, g; 1 mm in c; 500 µm in d. Abbreviations: ca, carapace; gi, gills; he, hepatopancreas; oe, oesophagus; pa, prehensile appendage; st, stomach; sw, stomach wall; tu, tubule; va, valve; vp, ventral pouch.

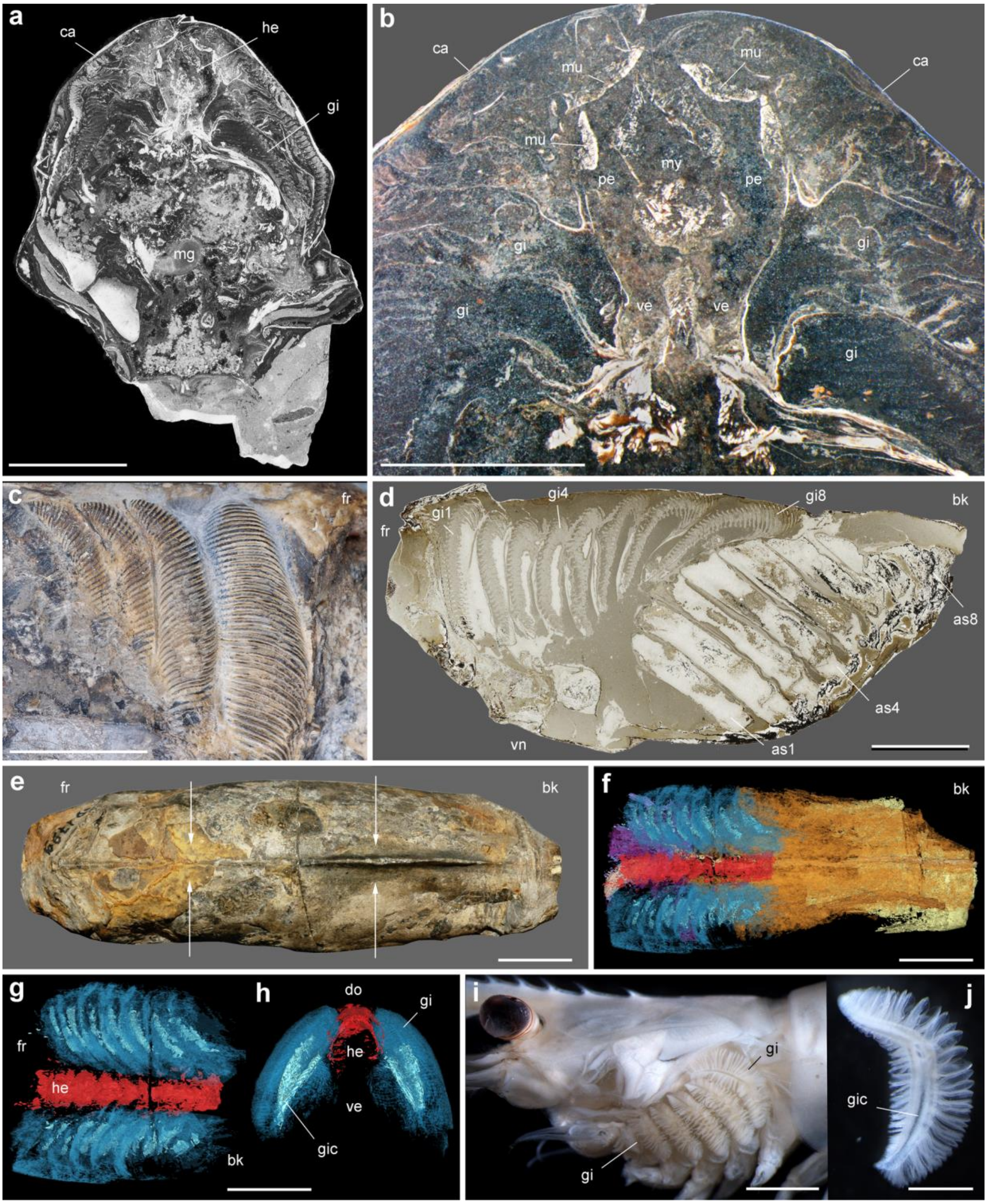

Supplementary Figure 2 | Cardio-vascular and respiratory systems of *Dollocaris ingens* (Middle Jurassic La Voulte Lagerstätte). **a,b**, MNHN.F.R06054, polished transverse section through the heart, general view and details. **c**, MNHN.F.R50937, three-dimensionally preserved gills. **d**, MNHN.F.A53004, longitudinal thin section through gills and abdominal segments. **e-h**, UJF-ID-1799, dorsal view and XTM images showing two symmetrical series of 8 gills (blue) ob both sides of the heart (red); location of gills indicated by white arrows. **i, j,** *Crangon crangon* (Recent), dissected specimen with exposed phyllobranchiate gills, general view and details of a gill with afferent and efferent canals. Scale bars: 1 cm in a, c-h; 5 mm in b; 2 mm in i, j. Abbreviations: as1-8, abdominal segments 1 to 8; bk, back; ca, carapace; do, dorsal; fr, front; gi, gill; gi1-8, 1st to 8th pair of gills; gic, gill canal; he, heart; mg, midgut; mu; muscles; my, myocardium; pe, pericardium; ve, vessel; vn, ventral;

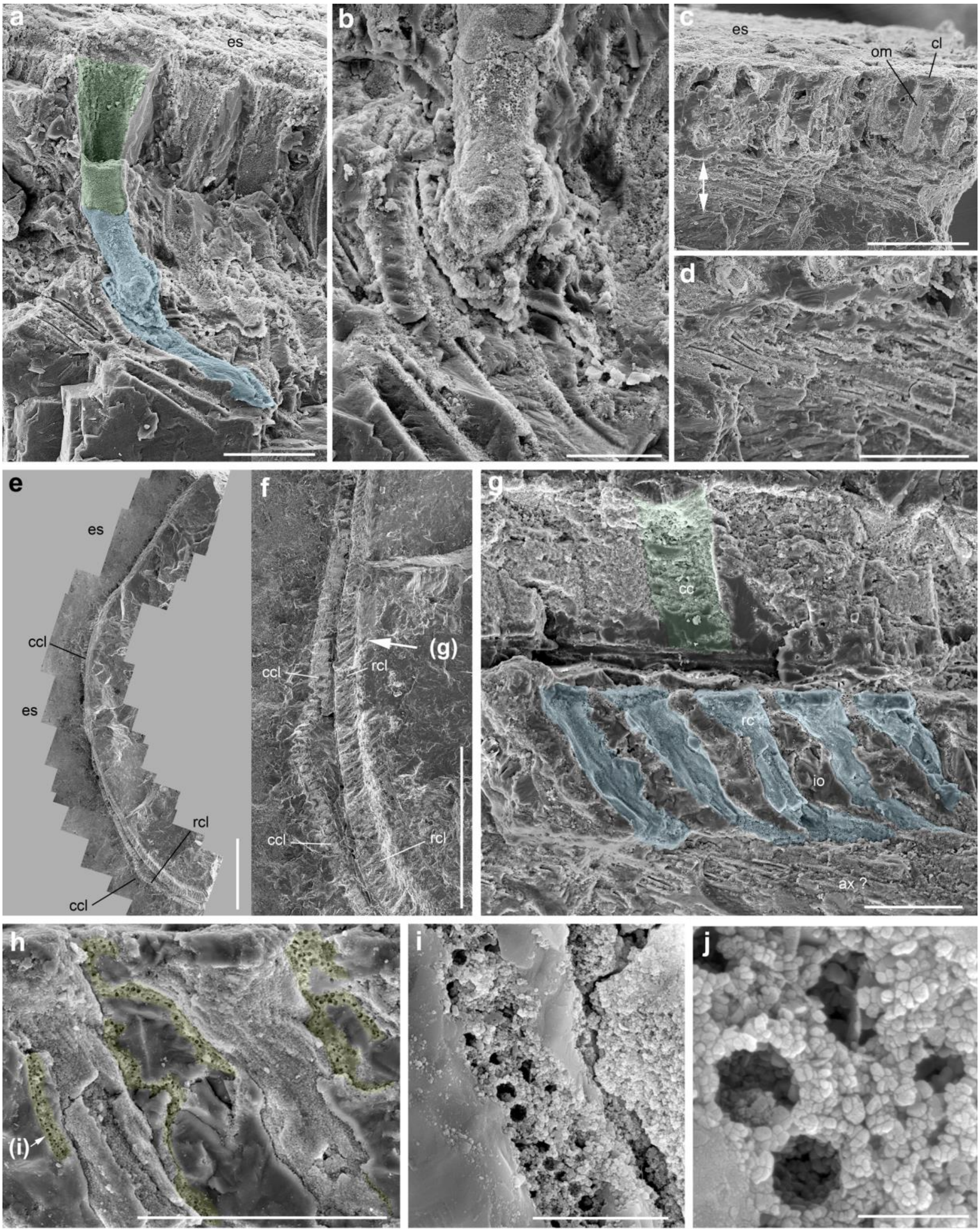

Supplementary Figure 3 | Internal eye structure of thylacocephalans exemplified by *Dollocaris ingens* (Middle Jurassic La Voulte Lagerstätte), FSL 710064. **a, b**, ommatidium (crystalline cone in green, retinula cells in blue), general view and details; . **c, d**, longitudinal section through ommatidia showing a basal fibrous layer (white arrow) that possibly represents axonal structures, general view and details. **e-j**, section through eye, general view and details of area show in g; crystalline cones layer slightly dissociated from the retinula cells layer. **g**, ommatidia in longitudinal section (crystalline cone in green, retinula cells in blue); note underlying oblique (possibly axonal) structures. **h-j**, small spherical hollows along the external boundary of retinula cells; general view showing their distribution (yellow) and details. Scale bars: 1 mm in e; 500 µm in f; 100 µm in c; 50 µm in a, d, g, h; 20 µm in b; 5 µm in i; 1 µm in j.

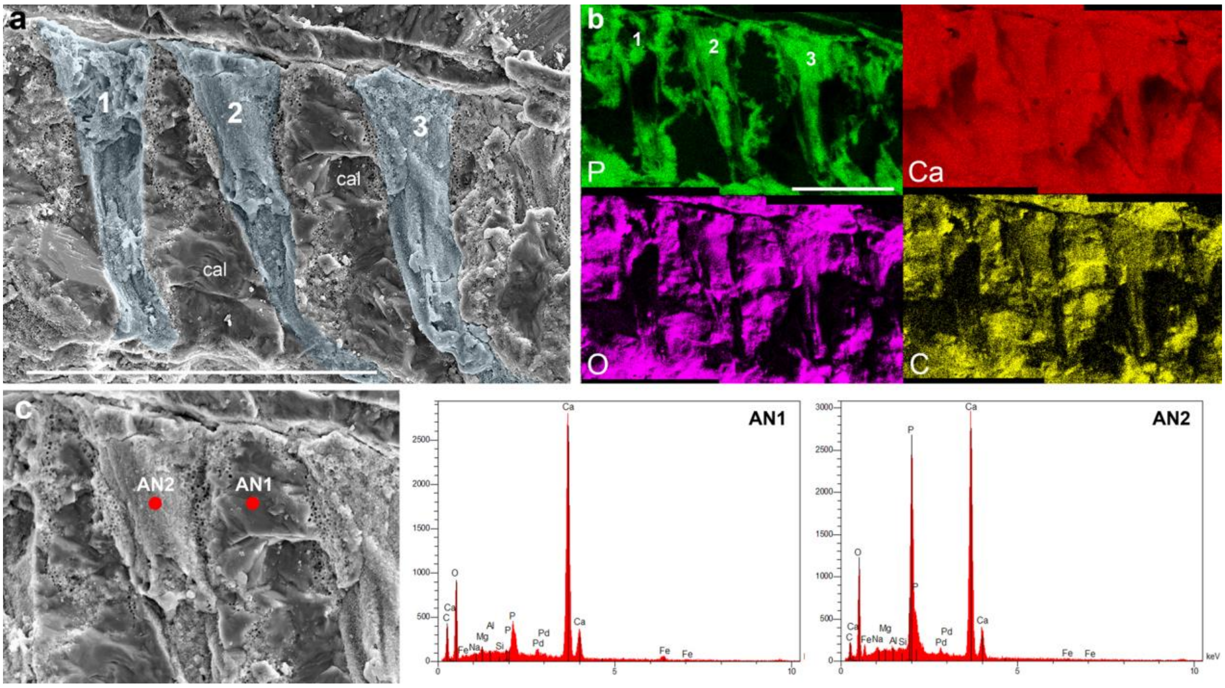

Supplementary Figure 4 | Preservation of the eye structure in the thylacocephalan *Dollocaris ingens* from the Middle Jurassic of the La Voulte Lagerstätte), FSL 710064. **a, b**, elemental mapping (EDX) showing the phosphatic composition (apatite) of the retinula cells region and the calcitic composition of the inter-ommatidial area. c, spot EDX analyses. Abbreviations: AN1, analysis 1 in inter-ommatidial area; AN2, analysis 2 in retinula cells; C, Carbon; Ca, Calcium; cal, calcite; O, Oxygen; P, Phosphorus; 1-3, three ommatidia. Scale bars: 100 µm in a; 50 µm in b.

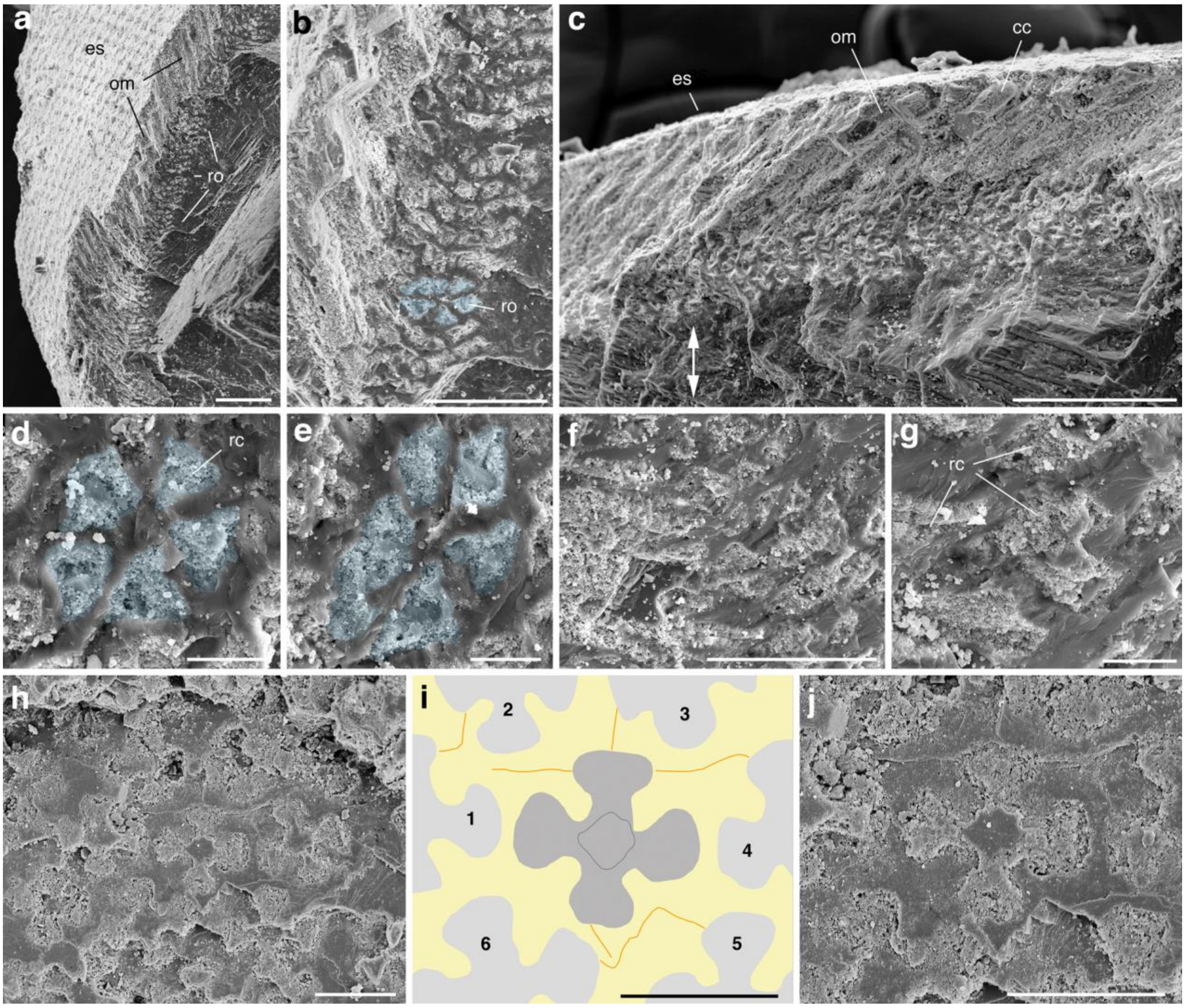

Supplementary Figure 5 | Internal eye structure of thylacocephalans exemplified by *Dollocaris ingens* (Middle Jurassic La Voulte Lagerstätte). **a-c**, section through the retinula cells of ommatidia with rosette-like structures (blue). **d, e**, transverse section through ommatidia showing rosettes with five retinula cells. **f, g**, oblique sections through retinula cells. h-i, general view of a transverse section through ommatidia, details showing one ommatidium (section through retinula cells) with six adjacent ommatidia and the surrounding inter-ommatidial space in pale yellow (possibly pigmented cells; possible boundaries in orange). Abbreviations: cc, crystalline cone; cl, corneal lens; es, eye external surface; om, ommatidium; rc, retinula cell. FSL 710064 in a-g; MNHN.F.R06206 in h-j. All SEM images. Scale bars: 100 µm in a, c; 50 µm in b, f, h-j; 10 µm in d, e, g.

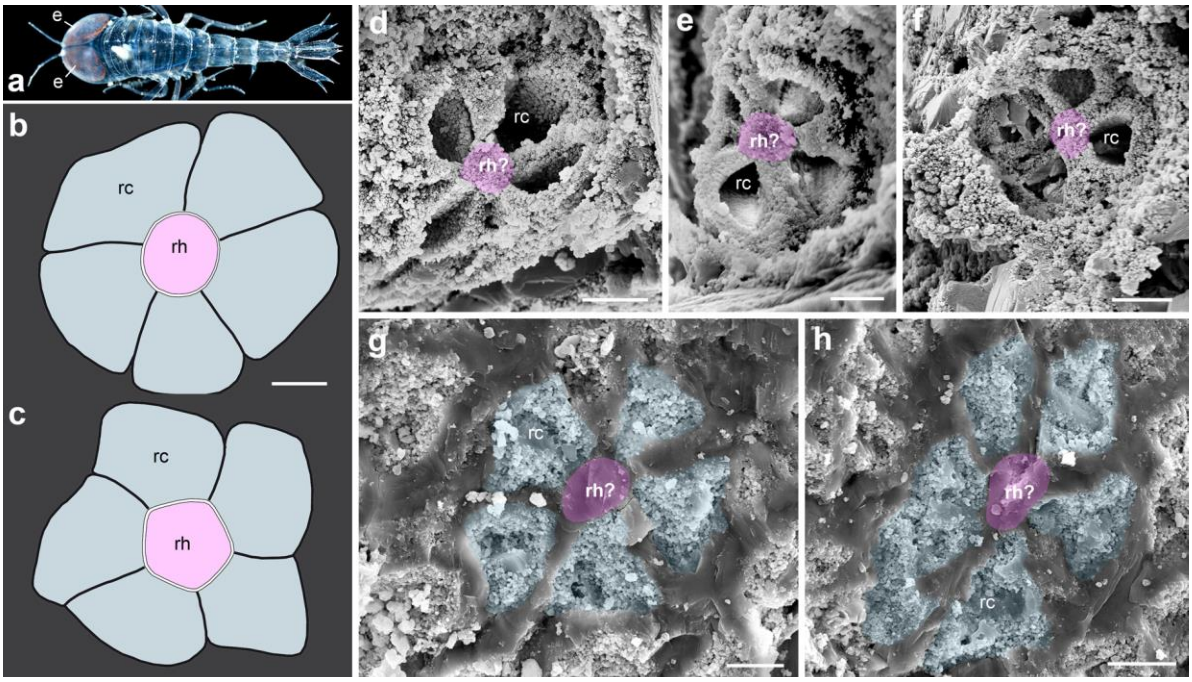

Supplementary Figure 6 | Comparison between the receptor cells in the ommatidium of Recent hyperiid (Amphipoda) crustacean and the thylacocephalan *Dollocaris ingens* from the Middle Jurassic of the La Voulte Lagerstätte). **a**, dorsal view of the deep-sea hyperiid *Phronima* (courtesy K.J. Osborne, Smithsonian Institution). **b, c,** simplified drawing of transverse sections through the ommatidium of *Phronima*[9] (from Transmission Electron Microscope images; figs. 16, 17) showing radially arranged retinula cells (blue) and central rhabdom (pink). **d-h**, FSL 710064, rosette-like structures (transverse sections through retinula cells clusters) in *Dollocaris*. Abbreviations: e, eye; rh, rhabdom; rh?, possible rhabdom (no clear boundaries); rc, retinula cell. Scale bars: 5 µm in b-h.

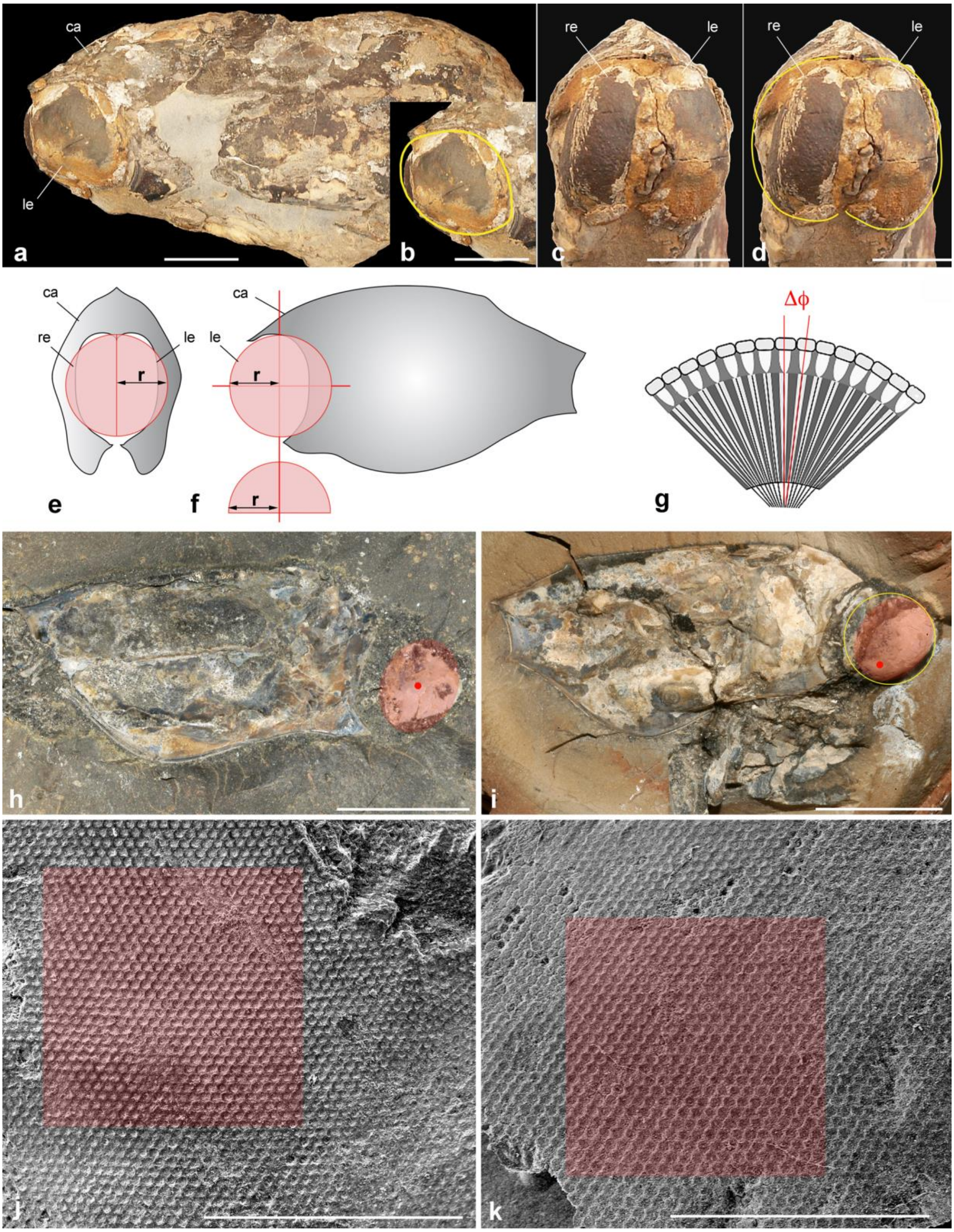

Supplementary Figure 7 | Eye shape and facet density in the thylacocephalan *Dollocaris ingens* from the Middle Jurassic of the La Voulte Lagerstätte. **a-d**, MHNL-20293244 showing a pair of hemispherical eyes (outline in yellow). **e, f**, simplified diagram which consider that the eye is hemispherical. **g**, simplified diagram through ommatidia to define the inter-ommatidial angle ($\Delta\phi$). **h, j**, MNHN.F.A29278, general view and details of hexagonal facets (red area equals 1 mm^2; location indicated by a red spot in h). **i, k**, FSL 710064, general view and details of hexagonal facets (red area equals 1 mm^2; location indicated by a red spot in e). Abbreviations: ca, carapace; $\Delta\phi$, inter-ommatidial angle; le, left eye; re, right eye. Scale bars: 10 mm in a-d, h, i and 1 mm in j, k.

species	classification	env.	age	N/eye	D (μm)	Δϕ (°)	AC	S	references
Dollocaris ingens	crustacean	?	Jurassic	18000-21000	40	1	1	nd	present study
Anax junius	dragonfly (Insecta)	T	Recent	30000	60[a]	0.25[a]	4	nd	Land and Nilsson 2002
Daphnia	crustacean (Branchiopoda)	SW	Recent	22	35	38	0.026	13.7	Land 1984; Young and Downing 1976
Artemia	crustacean (Branchiopoda)	SW	Recent	300	20	9	0.11	0.5[b] - 2.3[c]	Land 1984; Criel 1991
Cirolana	crustacean (Isopoda)	DW	Recent	60	100-150	15	0.067	6410	Land 1984; Nilsson 1983
Phronima	crustacean (Amphipoda)	MW	Recent	750	135-185[d]	0.4[d]	2.5	21[d]	Land 1981, 1984, 1989
Leptograpsus	crustacean (Decapoda)	SH	Recent	several 1000	45	1.5	0.67	0.7[b]-3.7[c]	Land 1984, Warrant 2006
Limulus	chelicerate (Xiphosura)	SW-NH	Recent	ca 850	200 - 300	8	0.12	127[b] - 1190[c]	Land 1984

Supplementary Table 1 | Measurements and visual parameters in the thylacocephalan *Dollocaris ingens* (Middle Jurassic, La Voulte Lagerstätte) and Recent crustaceans, insects and chelicerates. Abbreviations : AC, acuity; D, rhabdom diameter; DW, deep-water; Env, environment; MD, mid-water; N, approx. number of ommatidia; Nd, no data; NH, nocturnal habits; S, sensitivity; SH, shore; SW, shallow-water; T, terrestrial; Δϕ, inter-ommatidial angle; [a], in dorsal acute zone; [b], light-adapted; [c], dark-adapted; [d], dorsal eye; References (1)-(9): see Supplementary References.

Supplementary Note 1 | Measurements and calculation of eye parameters in *Dollocaris ingens* (Middle Jurassic La Voulte Lagerstätte).

In the majority of cases, the eyes of *Dollocaris* are crushed, fragmented or displaced with ommatidia being frequently bent (Supplementary Fig. 3g). Very few three-dimensionally preserved eyes were suitable for accurate measurements of their mineralized internal structures. Only two specimens (FSL 710064 and MNHN.F.A29278) offered optimal conditions for measuring locally the number of ommatidia and their density.

1-Ommatidial density. Facet density was measured from SEM images taken from the right eye of FSL 710064 and MNHN.F.A29278 (Supplementary Fig. 7).

Specimen FSL 710064 - The selected 1 mm^2 area has ca 25x20 = 500 ommatidia (Supplementary Fig. 7k).

Specimen MNHN.F.A29278 - The selected 1 mm^2 area has ca 27x23 = 621 ommatidia (Supplementary Fig. 7j).

2-Number of ommatidia (N), estimated inter-ommatidial angle ($\Delta\phi$)

Our fossil specimens do not allow direct measurements of $\Delta\phi$. For simplification, each eye was considered as an hemisphere (radius= r; Supplementary Fig. 7e, f) its surface area (A) being $2\pi r^2$. Because the eyes of *Dollocaris* are sunk into optical notches we assumed that between 10 and 20% of the surface area are devoid of facets and visual units. We used the method proposed by Land (1997) to estimate $\Delta\phi$ as follows: "An insect eye that covers 180° of space has a total field of view containing 20,626 square degrees (a solid angle 1° high and 1° wide). A hexagonal field of view covers $0.866\Delta\phi^2$ square degrees, where $\Delta\phi$ is the angular separation of centers of the hexagons. Thus the number (N) of ommatidial fields of view that can cover an hemisphere, without overlap is $23818/\Delta\phi^2$".

Specimen FSL 710064 - Total length (L) including carapace and eye = 34.6 mm; approximate radius of eye (r; see yellow outline; Supplementary Fig. 7i)= 2.7 mm. A= 45.78 mm^2. Since we have ca 500 hexagonal facets per mm^2 (Supplementary Fig. 7k), N ranges between 18315 (20% of A with no visual units) and 20600 (10%) ommatidia per eye.

3-Rhabdom acceptance angle ($\Delta\rho$)

$\Delta\rho$ is the angle subtended by the rhabdom tip at the nodal point of the corneal lens. An approximation to $\Delta\rho$ is given by $\Delta\rho^2 = (l/D)^2 + (d/f)^2$ (Supplementary references, ref. 2), where *l* is the wave length (0.5 µm), *D* the facet diameter, d the diameter of the rhabdom and f the focal length. None of our specimens allows the rhadom diameter to be measured thus precluding the calculation of $\Delta\rho$.

Supplementary references

1. Land, M.F., Optics and Vision in *Invertebrates in Vision in invertebrates (Handbook of Sensory Physiology, vol. 7/6B)* edited by H. Autrum (Springer, Berlin, 1981), pp. 471-492.
2. Land, M.F., Optics of the eys of *Phronima* and other deep-sea amphipods. *Journal of Comparative Physiology* 145, 209-226 (1981).
3. Land, M.F., The eyes of hyperiid amphipods : relations of optical structure to depth. *Journal of Comparative Physiology* 164, 751-762 (1989).
4. Land, M.F. & Nilsson, D.-E., *Animal eyes*. Oxford University Press, Oxford (2002).
5. Land, M.F. 1984., Crustacea in *Photoreception and vision in invertebrates* edited by M.A. Ali (Plenum, 1984), pp. 401-438.
6. Young, S. & Downing, A.C., The receptive fields of Daphnia ommatidia. *Journal of Experimental Biology* 64, 185-202 (1976).
7. Nilsson, H.L., Fine structure and convergent development of the *Cirolana* compound eye (Crustacea, Isopoda). *Zoomorphology* 102, 165-174 (1983).
8. Warrant, E.J., Invertebrate vision in dim light in *Invertebrate Vision* edited by E. J. Warrant & D.-E. Nilsson (Cambridge University Press, Cambridge, 2006), pp. 83-126.
9. Ball, E.E., Fine structure of the compound eyes of the midwater amphipod *Phronima* in relation to behavior and habitat. *Tissue and Cell* 9, 521-536 (1977).
